# Prevalence and Disease Spectrum of Extracoronary Arterial Abnormalities in Spontaneous Coronary Artery Dissection

**DOI:** 10.1001/jamacardio.2021.4690

**Published:** 2021-11-24

**Authors:** Alexandre Persu, Marilucy Lopez-Sublet, Abtehale Al-Hussaini, Marco Pappaccogli, Ibtissem Radhouani, Patricia Van der Niepen, William Adair, Christophe Beauloye, Pierre-Yves Brillet, Nathan Chan, Patrick Chenu, Hannes Devos, Javier Escaned, Marcos Garcia-Guimaraes, Frank Hammer, Robert Jackson, Salma Jebri, Deevia Kotecha, Fernando Macaya, Ciara Mahon, Nalin Natarajan, Kandiyil Neghal, Edward D. Nicol, Kelly S. Parke, Diluka Premawardhana, Averachan Sajitha, Joanne Wormleighton, Nilesh J. Samani, Gerry P. McCann, David Adlam

**Affiliations:** 1 Division of Cardiology, Cliniques Universitaires Saint-Luc, Université Catholique de Louvain, Brussels, Belgium; 2 Pole of Cardiovascular Research, Institut de Recherche Expérimentale et Clinique, Université Catholique de Louvain, Brussels, Belgium; 3 Department of Internal Medicine, ESH Hypertension Excellence Centre, CHU Avicenne, AP-HP, Bobigny, France; 4 INSERM UMR 942 MASCOT, CHU Avicenne, AP-HP, Bobigny, France; 5 Department of Cardiovascular Sciences, University of Leicester, NIHR Leicester Biomedical Research Centre, Glenfield Hospital, Leicester, United Kingdom; 6 Division of Internal Medicine and Hypertension Unit, Department of Medical Sciences, University of Turin, Turin, Italy; 7 Department of Radiology, CHU Avicenne, AP-HP, UMR INSERM U1272, Université Sorbonne Paris Nord, Bobigny, France; 8 Department of Nephrology & Hypertension, Universitair Ziekenhuis Brussel, Vrije Universiteit Brussel, Brussels, Belgium; 9 University Hospitals of Leicester, Glenfield Hospital, Leicester, United Kingdom; 10 Department of Radiology, Universitair Ziekenhuis Brussel, Vrije Universiteit Brussel, Brussels, Belgium; 11 Hospital Clínico San Carlos, IdISSC, Universidad Complutense, Madrid, Spain; 12 Department of Cardiology, Hospital del Mar, Parc de Salut Mar, Barcelona, Spain; 13 Division of Radiology, Cliniques Universitaires Saint-Luc, Université Catholique de Louvain, Brussels, Belgium; 14 Royal Brompton and Harefield NHS Foundation Trust London, London, United Kingdom; 15 National Heart and Lung Institute, Imperial College, London, United Kingdom

## Abstract

**Importance:**

Spontaneous coronary artery dissection (SCAD) has been associated with fibromuscular dysplasia (FMD) and other extracoronary arterial abnormalities. However, the prevalence, severity, and clinical relevance of these abnormalities remain unclear.

**Objective:**

To assess the prevalence and spectrum of FMD and other extracoronary arterial abnormalities in patients with SCAD vs controls.

**Design, Setting, and Participants:**

This case series included 173 patients with angiographically confirmed SCAD enrolled between January 1, 2015, and December 31, 2019. Imaging of extracoronary arterial beds was performed by magnetic resonance angiography (MRA). Forty-one healthy individuals were recruited to serve as controls for blinded interpretation of MRA findings. Patients were recruited from the UK national SCAD registry, which enrolls throughout the UK by referral from the primary care physician or patient self-referral through an online portal. Participants attended the national SCAD referral center for assessment and MRA.

**Exposures:**

Both patients with SCAD and healthy controls underwent head-to-pelvis MRA (median time between SCAD event and MRA, 1 [IQR, 1-3] year).

**Main Outcome and Measures:**

The diagnosis of FMD, arterial dissections, and aneurysms was established according to the International FMD Consensus. Arterial tortuosity was assessed both qualitatively (presence or absence of an S curve) and quantitatively (number of curves ≥45%; tortuosity index).

**Results:**

Of the 173 patients with SCAD, 167 were women (96.5%); mean (SD) age at diagnosis was 44.5 (7.9) years. The prevalence of FMD was 31.8% (55 patients); 16 patients (29.1% of patients with FMD) had involvement of multiple vascular beds. Thirteen patients (7.5%) had extracoronary aneurysms and 3 patients (1.7%) had dissections. The prevalence and degree of arterial tortuosity were similar in patients and controls. In 43 patients imaged with both computed tomographic angiography and MRA, the identification of clinically significant remote arteriopathies was similar. Over a median 5-year follow-up, there were 2 noncardiovascular-associated deaths and 35 recurrent myocardial infarctions, but there were no primary extracoronary vascular events.

**Conclusions and Relevance:**

In this case series with blinded analysis of patients with SCAD, severe multivessel FMD, aneurysms, and dissections were infrequent. The findings of this study suggest that, although brain-to-pelvis imaging allows detection of remote arteriopathies that may require follow-up, extracoronary vascular events appear to be rare.

## Key Points

**Question:**

What is the prevalence of fibromuscular dysplasia, aneurysms, dissection, and tortuosity in extracoronary arteries of patients who developed a spontaneous coronary artery dissection (SCAD)?

**Findings:**

In this case series including 173 patients with SCAD, using magnetic resonance angiography with blinded interpretation of the findings, 32% of the patients had fibromuscular dysplasia, 8% had aneurysms, and 2% had dissections; the prevalence of arterial tortuosity was similar in cases and controls. Extracoronary vascular events over a median 5-year follow-up were rare.

**Meaning:**

The findings of this blinded analysis suggest that, in patients with SCAD, severe multivessel fibromuscular dysplasia, aneurysms, and dissections are infrequent and seldom associated with clinically evident vascular events.

## Teaser

This case series examines the prevalence of fibromuscular dysplasia and other extracoronary events in patients who develop spontaneous coronary artery dissection.

## Introduction

Spontaneous coronary artery dissection (SCAD) is a cause of acute coronary syndrome predominantly affecting young to middle-aged women, including a minority during or after pregnancy. SCAD is characterized by the development of a false lumen within the coronary artery wall that leads to coronary insufficiency due to external compression of the true lumen. This event has the potential to cause myocardial infarction.^1,2^ Although SCAD has historically been considered a very rare diagnosis, its prevalence has been recently reevaluated, and SCAD is now estimated to cause 10% to 25% of acute coronary syndrome presentations in women younger than 50 years.^3,4^

SCAD has been associated with various extracoronary arteriopathies.^5^ The most frequent of these is fibromuscular dysplasia (FMD), a nonatherosclerotic, noninflammatory disease of arterial walls that also occurs predominantly in middle-aged women and shares a common genetic risk variant with SCAD.^6^ The reported prevalence of FMD in SCAD ranges widely, from 11% to 86%.^1,2^ Lesions were reported to be almost exclusively the multifocal string-of-beads subtype and have been identified most commonly in renal, cerebrovascular, and iliac arteries.^7,8^ In addition to typical FMD lesions, other extracoronary vascular abnormalities, such as aneurysms, dissections, irregularities, undulations, and/or arterial tortuosity, have been reported.^7^ Based on these findings, both the SCAD^1,2^ and FMD^9,10^ consensus statements recommend brain-to-pelvis imaging by computed tomographic angiography (CTA) or magnetic resonance angiography (MRA) at least once.

To date, studies assessing the prevalence and type of extracoronary lesions in patients with SCAD have had important limitations. First, the diagnostic criteria for FMD, arterial tortuosity, and other vascular abnormalities have not always been clearly defined. Second, all previous studies lacked a control group or blinded analysis. Third, assessment of severity and quantification of extracoronary arterial lesions have not been provided. Therefore, some investigators may have reported only the typical string-of-beads lesions, while others may have also considered mild arterial irregularities as evidence of FMD.

The objectives of this blinded study were to assess the prevalence, type, and severity of FMD lesions and other extracoronary arterial abnormalities in patients with SCAD and quantify extracoronary artery tortuosity in this population.

## Methods

### Study Participants

Patients from the UK SCAD Registry were invited to participate in the SCAD Deep Phenotyping Study. Patients with angiographically confirmed SCAD in the registry were recruited between January 1, 2015, and December 31, 2019 (median time between SCAD event and MRA, 1 [IQR, 1-3] year) throughout the UK by referral from the clinical team at the presenting hospital, primary care referral, or self-referral to an online web portal. The study protocol was approved by the UK National Research Ethics Service and the UK Health Research Authority and conducted in accordance with the Declaration of Helsinki.^11^ All participants gave fully informed and signed consent; there was no financial compensation. The study followed the reporting guideline for case series.

Of 315 eligible patients recruited to the UK SCAD registry, 192 consented to undergo phenotyping. Of these, 1 patient died before imaging was conducted, 3 patients had implantable cardioverter defibrillators, 1 patient had claustrophobia, 5 were excluded on angiographic grounds as not meeting the study criteria for definite SCAD, 8 individuals were excluded due to technical issues preventing complete or adequate quality scanning, and 1 patient subsequently declined to attend despite initially consenting (eFigure in the Supplement). Therefore, 173 patients (55%) from the Registry were included in this analysis. Participants attended the national SCAD referral center for assessment and MRA imaging. A subset of patients also underwent CTA screening assessment as part of routine clinical care of SCAD with scans undertaken at 2 UK SCAD national centers (Leicester and London [Royal Brompton/Chelsea and Westminster]).

Medical records and imaging results from the index SCAD event and subsequent potential major adverse cardiovascular and cerebrovascular events were obtained. Basic demographic information collected included self-reported race and ethnicity because it is not known whether the prevalence and spectrum of extracoronary arteriopathies in patients with SCAD differs between racial and ethnic groups. A detailed medical history and clinical examination, including determination of a Beighton score (a simple 9-point clinical score to assess hypermobility and joint laxity, with higher scores indicating greater hypermobility and joint laxity),^12^ was obtained at the time of the patient visit. Pregnancy-associated SCAD was defined as an event occurring during gestation or within 12 months of delivery.

Healthy individuals serving as controls were recruited by open advertisement and targeted to match the age and sex profile of the SCAD cohort. This control cohort was used for interpretation of MRA images by investigators blinded to participants’ status. These control participants were screened to exclude those with prior hypertension to reduce the likelihood of including individuals with potential renovascular disease.

All angiographic analysis was conducted with investigators blinded to the results of the MRA/CTA analysis. All patients had their SCAD diagnosis confirmed by 2 experienced SCAD clinicians (A.A.-H. and M.G.-G.) with adjudication of any differences by a third experienced SCAD clinician (D.A.). Patients with atherosclerotic, traumatic, or iatrogenic dissection (except in the context of definite SCAD) were excluded.

### Genetic Testing

Genotyping at the *PHACTR1* locus rs9349379 was performed by assay as previously described (TaqMan; ThermoFisher Scientific)^6^ in 114 patients with SCAD and 49 healthy controls.

### Imaging

Patients with SCAD and healthy controls underwent research MRA scanning using a common protocol on a 3-T platform (Siemens Avanto). Imaging of the head and neck vessels was performed using time-of-flight noncontrast MRA sequences. Whole aorta MRA with iliac/femoral arteries was performed using a 3-dimension T1 gradient echo sequence, with parallel imaging. Gadoterate meglumine, 0.1 mmol/kg, was administered in a single injection at 4 mL per second followed by a 20-mL saline flush at the same speed.

Computed tomographic angiography was performed using a standard protocol (Somatom Definition Flash; Siemens Healthineers). Scan range was from the circle of Willis to the femoral heads. A biphasic injection protocol was used with 100 mL of iodinated contrast and a 100-mL bolus of sodium chloride injected at 2 mL per second. To ensure adequate opacification of the entire arterial system, bolus tracking was used (with a region of interest placed in the aortic arch) with a 7-second delay. Raw data were reconstructed using 1-mm contiguous slices for subsequent analysis.

All anonymized MRA and CTA images were jointly analyzed by a vascular medicine specialist (M.L.-S.) and a vascular radiologist (I.R.) using a detailed preestablished case report form, with adjudication of any differences by a third FMD specialist (A.P.). All 3 specialists were unaware of the participant’s clinical status (healthy control vs SCAD), the number of controls, and any other demographic or disease-related characteristics. Magnetic resonance angiography and CTA from the same patient were unlinked and analyzed independently.

### Extracoronary Arterial Abnormalities

Analysis was undertaken in a single center, using viewing software (Carestream Vue PACS; Carestream Health Inc). Analysis included cerebrovascular, renal, visceral, and iliofemoral vascular beds as well as the thoracoabdominal aorta. The following lesions were considered: multifocal stenosis (string of beads, ie, the presence of alternating areas of stenosis and dilatation), focal stenosis, arterial aneurysms, and dissections. Aneurysm was defined as the presence of greater than 50% enlargement in the diameter of an artery in the orthogonal plane or perpendicular to the long axis of the vessel compared with an adjacent normal arterial segment. The diagnosis of FMD was based on the identification of a string of beads in at least 1 arterial bed. Multivessel FMD was defined as the presence of a string of beads in at least 2 different arterial beds or a string-of-beads in 1 vascular bed and aneurysm or dissection in 1 or more vascular beds in accordance with the international consensus on FMD.^9,10^

Arterial tortuosity was assessed by the number of angulations greater than or equal to 45° in the carotid, vertebral, renal, and iliac arteries. In cases of 1 or more loops greater than or equal to 45°, tortuosity index ([direct length of the vessel/straightened vessel length −1] × 100)^13^ was also calculated.^14^ For the calculation of arterial length, the same anatomical references were used for each measurement (vertebral artery: from origin to second cervical vertebra^15^; internal carotid: from carotid bifurcation to horizontal segment; and renal, common, and external iliac arteries: from the ostium to first bifurcation).

### Statistical Analysis

All analysis was performed using SPSS, version 21.0 (IBM Corp). Continuous variables are expressed as mean (SD) or median (IQR) according to their distribution; categorical variables are expressed as counts and percentage. Data were analyzed with the Kolmogorov-Smirnov test to determine their distribution. Continuous variables were analyzed using the unpaired *t* test if distribution was gaussian or the Mann-Whitney test in case of nongaussian distribution. Categorical variables were compared using the χ^2^ test. Statistical significance was set at 2-sided *P* value <.05 for all analysis.

## Results

### Characteristics of Patients With SCAD vs Controls

Clinical characteristics of patients with SCAD and healthy controls are reported in Table 1. Magnetic resonance angiography analysis was undertaken on 173 patients with SCAD and 41 controls with investigators blinded to the clinical diagnosis. The SCAD cohort comprised 167 women (96.5%) and 6 men (3.5%); mean (SD) age at diagnosis was 44.5 (7.9) years. Of 170 participants with race and ethnicity data known, the categories comprised Black African and/or Caribbean (2 [1.2%]), Indian (5 [2.9%]), and White (163 [94.2%]); 2 participants reported Other (2 [1.2%]) and 1 did not report data. Risk factors for vascular disease included smoking (49 [28.3%]), hypertension (31 [17.9%]), and dyslipidemia (14 [8.1%]). The control group was also predominantly female (40 [97.6%]) but slightly younger (41.6 [7.4] years) and more ethnically diverse than the SCAD cohort (Table 1).

**Table 1.** Demographic and Clinical Characteristics of Patients With Spontaneous Coronary Artery Dissection vs Controls

Characteristic	No. (%)	
	Patients with SCAD (n = 173)	Controls (n = 41)^a^
Sex		
Women	167 (96.5)	40 (97.6)
Men	6 (3.5)	1 (2.4)
Age at diagnosis, mean (SD), y	44.5 (7.9)	41.6 (7.4)
BMI, mean (SD)	26.6 (10.6)	25.9 (6.0)
Smoking habit		
Never/former	124 (71.7)	26 (63.4)
Current	49 (28.3)	15 (36.6)
Race and ethnicity^b^		
Black African and/or Caribbean	2 (1.2)	1 (2.4)
Indian	5 (2.9)	2 (4.9)
White	163 (94.2)	35 (85.4)
Other	2 (1.2)	3 (7.3)
Treated hypertension	31 (17.9)	0
Type 2 diabetes	0	0
Treated dyslipidemia	14 (8.1)	0

Abbreviations: BMI, body mass index (calculated as weight in kilograms divided by height in meters squared); SCAD, spontaneous coronary artery dissection.

^a^ A control cohort was included to allow blinded interpretation of magnetic resonance angiograms by investigators who were blinded to SCAD status.

^b^ Race and ethnicity were unknown for 3 participants in the SCAD group; other was not specified by the participants.

### Characteristics of Patients With SCAD

SCAD was predominantly diagnosed following a non–ST segment elevation myocardial infarction (105 [60.7%]); 59 patients (34.1%) underwent percutaneous coronary intervention and 5 patients (2.9%) underwent coronary artery bypass grafting. The most frequently affected vessel was the left anterior descending coronary artery (122 [70.5%]). Twenty-seven patients (15.6%) had multivessel SCAD, 16 (9.2%) had experienced recurrent SCAD, and 15 of the episodes (9.0%) were associated with pregnancy (Table 2). Eighty-three patients (48.0%) reported migraines. The Beighton score was greater than 4 in 53 patients (30.6%). The distribution of genotypes at the *PHACTR1* locus (n = 163) was AA, 44.8% (n = 73); AG, 47.9% (n = 78); and GG, 7.4% (n = 12).

**Table 2.** Additional Characteristics of Patients With SCAD

Characteristic	Patients with SCAD, No. (%)
No.	173
Acute coronary syndromes at presentation of SCAD	
NSTEMI	105 (60.7)
STEMI	54 (31.2)
Cardiac arrest	14 (8.1)
PCI (n = 169)	59 (34.1)
Bailout CABG	5 (2.9)
Angiographic features	
Coronary artery involved	
Left main artery	12 (6.9)
Left anterior descending artery	122 (70.5)
Left circumflex artery	55 (31.8)
Right coronary artery	29 (16.8)
Portion of the artery involved	
Proximal	36 (20.8)
Middle	78 (45.1)
Distal	8 (50.9)
Branch	54 (31.2)
Multivessel	27 (15.6)
Multisegment	64 (37.0)
Saw class^a^	
1	24 (13.9)
2a	78 (54.5)
2b	32 (18.5)
3	15 (8.7)
4	23 (13.3)
Recurrence	16 (9.2)
SCAD occurring during peripartum (n = 167)	
During pregnancy	5 (3.0)
During postpartum	10 (6.0)

Abbreviations: CABG, coronary artery bypass graft; NSTEMI, non-ST elevation myocardial infarction; PCI, percutaneous coronary intervention; SCAD, spontaneous coronary artery dissection; STEMI, ST elevation myocardial infarction.

^a^Saw class categories: 1, multiple radiolucent lumens or arterial wall contrast staining; 2a, diffuse arterial narrowing bordered by normal segments; 2b, diffuse narrowing that extends to the distal tip of the artery; 3, focal or tubular stenosis; and 4, total occlusion, usually of a distal vessel.^1^

### Prevalence and Type of Extracoronary FMD and Other Arterial Abnormalities in Patients With SCAD

The prevalence of FMD was 31.8% (55 of 173) in the SCAD cohort. Among the 55 patients with SCAD and FMD, 70.9% (n = 39) had involvement of 1 arterial bed; 25.5% (n = 14), 2 arterial beds; and 3.6% (n = 2), 3 arterial beds. The most commonly affected arterial beds of the total 173 patients with SCAD were renal (27 [15.6%]), cervical (23 [13.3%]), iliac (17 [9.8%]), and visceral (5 [2.9%]) (Table 3). The prevalence of hypertension was not significantly different in patients with (3 of 27 [11.1%]) or without (28 of 146 [19.2%]) renal FMD (*P* = .32).

**Table 3.** Prevalence and Characteristics of Fibromuscular Dysplasia and Other Extracoronary Abnormalities in Patients With SCAD

Arterial abnormalities	No. (%)	
	Patients with SCAD (n = 173)	Controls (n = 41)
Fibromuscular dysplasia, arterial beds	55 (31.8)	1 (2.4)
1	39 (22.5)	1 (2.4)
2	14 (8.1)	0
≥3	2 (1.2)	0
**Prevalence of FMD lesions in each vascular bed**		
Renal	27 (15.6)	0
Cerebrovascular	23 (13.3)	0
Iliac	17 (9.8)	1 (2.4)
Visceral	5 (2.9)	0
**Aneurysms**		
Total	13 (7.5)	0
Renal	0	0
Cerebrovascular	1 (0.6)	0
Intracranial	3 (1.7)	0
Visceral	7 (4.0)	0
Iliac	1 (0.6)	0
Aortic	1 (0.6)	0
**Arterial dissections**		
Total	3 (1.7)	0
Renal	0	0
Cerebrovascular	2 (1.2)	0
Visceral	0	0
Iliac	1 (0.6)	0
Aorta	0	0
**Focal stenosis**		
Total	14 (8.7)	1 (2.4)
Renal	0	0
Cerebrovascular	6 (3.5)	0
Visceral (other than celiac trunk stenosis)	3 (1.7)	0
Celiac trunk	5 (2.9)	1 (2.4)
Iliac	0	0

Abbreviations: FMD, fibromuscular dysplasia; SCAD, spontaneous coronary artery dissection.

Focal stenosis in at least 1 arterial bed was reported in 15 patients (8.7%) with SCAD. Thirteen patients (7.5%) had aneurysms and 3 patients (1.7%) had dissections (Table 3). Subsets of patients with or without extracoronary FMD did not differ according to patient or SCAD-related characteristics. In particular, the distribution of *PHACTR1* genotypes did not differ between patients with SCAD with or without FMD (eTable 1 in the Supplement).

Irrespective of arterial beds, the prevalence of arterial tortuosity estimated by the number of loops greater than or equal to 45° or tortuosity index did not differ between patients and controls. Similarly, the prevalence of the carotid S curve was similar in both groups (right side: 5.2% vs 2.4%; *P* = .45; left side: 5.2% vs 7.3%; *P* = .60) (Table 4; eTable 2 in the Supplement). In contrast, arterial tortuosity in any arterial bed was significantly more frequent in hypertensive patients with SCAD (27 of 31 [87.1%]) compared with normotensive patients with SCAD (88 of 134 [65.7%]) (*P* = .02).

**Table 4.** Prevalence and Severity of Arterial Tortuosity in Cerebrovascular Vessels of Patients With SCAD vs Controls

Tortuosity evaluation^a^	Mean (SD)		*P* value
	Patients with SCAD (n = 173)	Controls (n = 41)	
**Cervical arteries**			
Right ECa			
≥1 Curve ≥45°, No./total No. (%)	63/172 (37)	13/41 (31.7)	.43
No. of curves ≥45°	2.1 (1.0)	1.5 (0.7)	.01
Mean angle	76.0 (36.5)	79.2 (41.8)	.79
S-curve, No./No. (%)	9/173 (5.2)	1/41 (2.4)	.45
Tortuosity index	18.9 (11.2)	14.6 (8.2)	.23
Left ECa			
≥1 Curve ≥45°, No./total No. (%)	64/173 (37.0)	11/41 (26.8)	.21
No. of curves ≥45°	1.9 (1.0)	1.6 (1.2)	.63
Mean angle	69.3 (22.7)	78.1 (20.5)	.32
S-curve, No./No. (%)	9/173 (5.2)	3/41 (7.3)	.60
Tortuosity index	18.5 (12.0)	19.6 (11.2)	.79
Right EVa			
≥1 Curve ≥45°, No./total No. (%)	70/173 (40.5)	10/41 (24.4)	.09
No. of curves ≥45°	2.2 (1.1)	2.0 (1.6)	.82
Mean angle	74.5 (23.8)	64.1 (19.6)	.31
Tortuosity index	11.0 (7.6)	10.4 (5.6)	.67
Left EVa			
≥1 Curve ≥45°, No./total No. (%)	80/173 (46.2)	18/41 (43.9)	.81
No. of curves ≥45°	2.5 (1.6)	1.9 (1.2)	.18
Mean angle	74.6 (18.3)	65.2 (23.3)	.09
Tortuosity index	12.4 (8.5)	11.5 (8.8)	.72

Abbreviations: ECa, extracranial carotid artery; EVa, extracranial vertebral artery; SCAD, spontaneous coronary artery dissection.

^a^ Tortuosity index was calculated in the subset with at least 1 curve greater than or equal to 45°.

### Prevalence of FMD and Other Arterial Lesions Assessed by CTA vs MRA

We subsequently compared the MRA assessment of remote arteriopathy prevalence through comparison with CTA assessment in 43 of 173 patients with SCAD in whom CTA arteriopathy screening had been conducted as part of routine clinical care in addition to paired research MRA. No significant difference was found in the prevalence, severity, and/or localization of FMD or arterial abnormalities as detected by either imaging modality (Figure and eTable 3 in the Supplement). The median dose length product for the CTAs was 710 (IQR, 419-745) mGy•cm.

**Figure.**
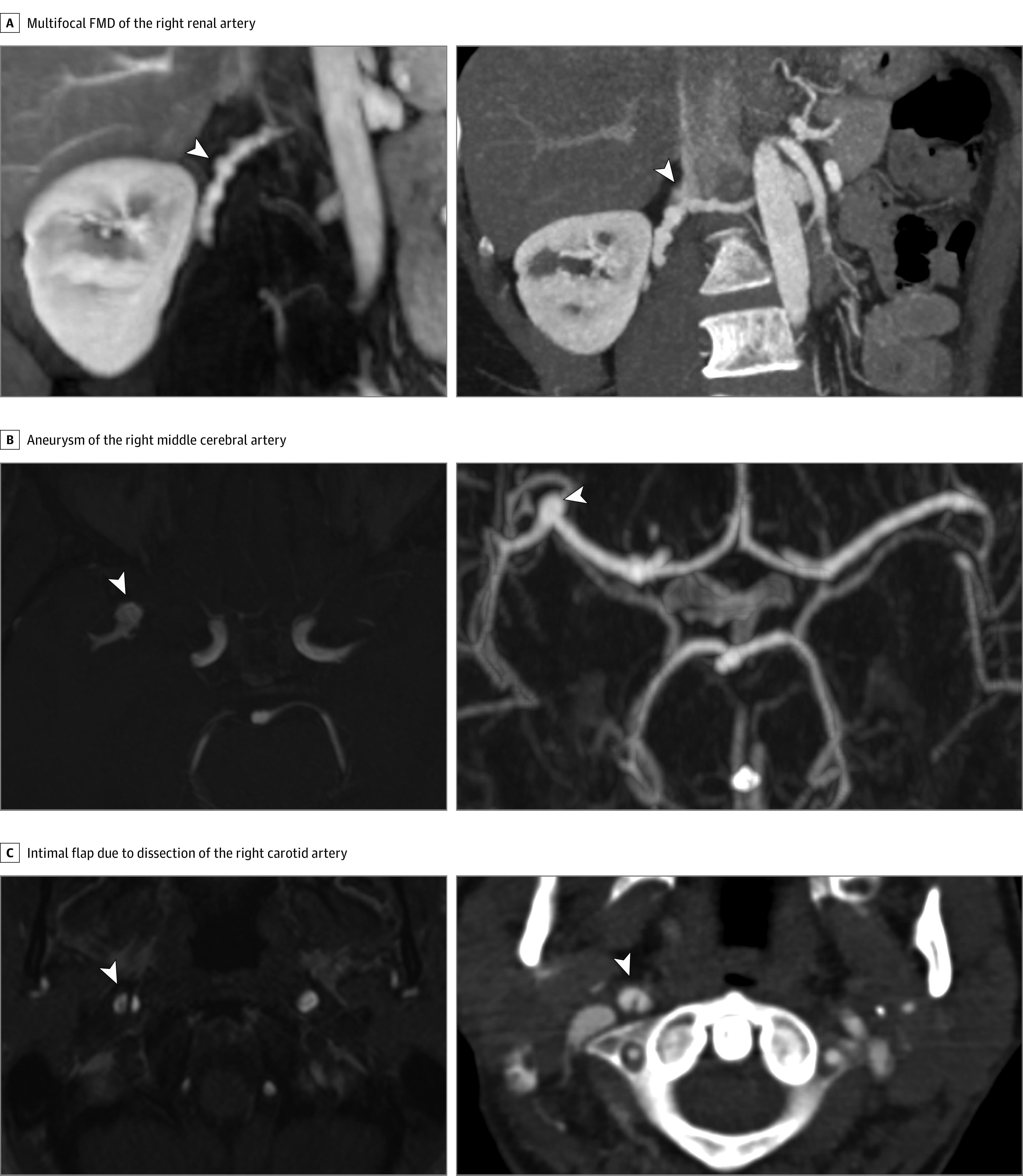
Examples of Extracoronary Arterial Abnormalities in 3 Patients With Spontaneous Coronary Artery Dissection Visualized by Computed Tomographic Angiography (CTA) and Magnetic Resonance Angiography (MRA) A, Multifocal fibromuscular dysplasia (FMD) (string of beads) of the mid-distal segments of the right renal artery. B, Aneurysm of the right middle cerebral artery (M1-M2 junction). C, Intimal flap due to a dissection of the right carotid artery. Lesions are indicated by arrowheads.

### Clinical Follow-up

Extracoronary vascular events (stroke, dissection, limb claudication, and revascularization of extracoronary arterial beds) were assessed in 170 of 173 patients at a median follow-up of 5 (IQR, 4-7) years after the index SCAD event. The remaining 3 patients were lost to detailed follow-up but are known to be alive. A single stroke event was reported (secondary to embolism of a left ventricular thrombus following recurrent SCAD). There were no other extracoronary vascular events or revascularization procedures. Major adverse events were reported in 41 of 170 patients with 2 deaths (both from noncardiovascular causes), 35 cases of recurrent myocardial infarction, and 4 cases of coronary revascularization.

## Discussion

To our knowledge, this study is the first blinded assessment of the prevalence and spectrum of FMD and other extracoronary arterial abnormalities in a large cohort of patients with SCAD. First, in this cohort assessed by MRA, we report a prevalence of FMD of 31.8%. Second, the prevalence of the most serious arterial abnormalities was 9.2% (aneurysms, 7.5%; dissections, 1.7%), which is substantially lower than that reported in noncontrolled, unblinded observational analyses of primary FMD.^16,17,18^ Third, the prevalence of multivessel involvement in patients with SCAD and FMD was 29.1% (2 arterial beds, 25.5%; 3 arterial beds, 3.6%), again much lower than that reported for patients with primary FMD.^9,10,16,17,18,19^ In addition, clinical sequelae from SCAD-related noncoronary arteriopathies were seldom observed.

According to a recent state-of-the art review by Hayes et al,^20^ the prevalence of extracoronary FMD in unblinded, uncontrolled studies including over 100 patients with SCAD was between 45.2% and 77.6%. In our blinded analysis strictly based on International FMD Consensus criteria,^9,10^ including both SCAD cases and healthy controls (which may reduce the risk of overdiagnosis), we reported a lower prevalence of 31.8%. Notably, this estimate is in keeping with a recently reported large Canadian SCAD cohort (n = 750) explored using various imaging modalities (52.4% CTA; 43.5% catheter-based angiography; and 4.0% MRA) in which the proportion of extracoronary FMD lesions was similar (ie, 31.1%),^21^ albeit from an incompletely screened population.

 In patients with SCAD and FMD, the prevalence of FMD in multiple vascular beds (multivessel FMD) was 29.1%, consistent with that documented by Prasad et al^7^ (29%), but lower than in previous SCAD studies by Saw et al^8^ (49%) and Liang et al^22^ (44%), as well as in patients with primary FMD included in large registries (57%-66%).^9,10,16,17,19^ Similarly, in this series of patients with SCAD, the prevalence of arterial aneurysms (7.5%) and dissections (1.7%) was much lower than that reported in registries of primary FMD (aneurysms: 21.6%-26.0%; dissections: 5.6%-28.1%).^16,17,18^ In addition, although arterial tortuosity is considered a manifestation of primary FMD,^9,10^ in our series of patients with SCAD, whether in the presence or absence of FMD, the prevalence of extracoronary arterial tortuosity was not higher than in healthy controls. In view of similar performance of MRA and CTA in 43 patients explored by both imaging modalities, these findings are unlikely to be explained by a lesser sensitivity of MRA vs CTA for detection of clinically relevant lesions.

Furthermore, the absence of an increased prevalence of hypertension in patients with SCAD and renal artery FMD supports the visual impression that most patients had mild renal FMD lesions, which are unlikely to have hemodynamic consequences. This finding is also in keeping with low rates of noncoronary vascular events reported in this and another study.^23^

Our findings lend further support to the concept that SCAD with FMD and primary FMD may correspond to distinct although overlapping entities. Along the same lines, dissections in patients with FMD are associated with the male sex^17,18,24,25^ and patients with SCAD are primarily female. Also, recent studies report only a partial overlap between genetic determinants of FMD and SCAD.^26^ In addition, a histological correlate for the radiological FMD in SCAD remains to be demonstrated.^27^ Further studies are needed to fully understand the interrelationship between SCAD and FMD.

### Limitations

This study has limitations; the first is the retrospective and cross-sectional design leading to the potential for bias. This was an observational study and therefore we cannot conclude that the documented associations are causative. Of 315 eligible patients recruited to the UK SCAD registry, only 192 consented to undergo phenotyping. However, the demographic and SCAD-related characteristics in our series are in line with those reported in other large SCAD cohorts.^7,21,28^ Second, the comparison of the prevalence of multivessel FMD, aneurysms, and dissections with that reported in registries focused on patients with primary FMD^16,17,18,19^ needs to be considered with caution. Indeed, in the latter, with the exception of the Assessment of Renal and Cervical Artery Dysplasia study,^16^ whole body scanning was not performed in all patients. Furthermore, data were registered by multiple investigators, central image analysis was not exhaustive, and analysis was limited to patients with FMD and therefore unblinded. Third, the size of the subgroup explored by both CTA and MRA was limited (n = 43), which reduces the power of the statistical comparisons presented. Larger studies will be needed to confirm our findings and assess smaller differences.

## Conclusions

In this blinded analysis of patients with SCAD, severe multivessel FMD, aneurysms, and dissections were infrequent. Although brain-to-pelvis imaging allows detection of remote arteriopathies which may require follow-up, supporting the current recommendation^1,2^ to perform a systematic arterial screening in all patients with SCAD, more research is required to assess whether this strategy leads to a reduction in clinical events. The findings of this study suggest that clinical complications from these extracoronary arterial lesions over the short to medium term appear to be very rare.

## Notes

Supplement. **eTable 1.** Comparison of General and Coronary Characteristics Between SCAD Patients With and Without FM **eTable 2.** Prevalence and Severity of Arterial Tortuosity in Renal and Iliac Vessels of Patients With Spontaneous Coronary Artery Dissection Versus Controls **eTable 3.** Prevalence and Characteristics of Fibromuscular Dysplasia and Other Extracoronary Abnormalities Detected by MRA and CTA in Patients Evaluated by Both Imaging Modalities **eFigure.** Flow Chart of Patients From the UK SCAD Registry Included in the Current Analysis
