# Supplementary material for: Prevalence and Disease Spectrum of Extracoronary Arterial Abnormalities in Spontaneous Coronary Artery Dissection
Source: JAMA Cardiol. 2021 Nov 24;7(2):159–66. doi: 10.1001/jamacardio.2021.4690 (PMC8613702; doi:10.1001/jamacardio.2021.4690)
Supplement: Supplement. — eTable 1. Comparison of General and Coronary Characteristics Between SCAD Patients With and Without FM eTable 2. Prevalence and Severity of Arterial Tortuosity in Renal and Iliac Vessels of Patients With Spontaneous Coronary Artery Dissection Versus Controls eTable 3. Prevalence and Characteristics of Fibromuscular Dysplasia and Other Extracoronary Abnormalities Detected by MRA and CTA in Patients Evaluated by Both Imaging Modalities eFigure. Flow Chart of Patients From the UK SCAD Registry Included in the Current Analysis [file jamacardiol-e214690-s001.pdf]

Supplementary Online Content

Persu A, Lopez-Sublet M, Al-Hussaini A, et al. Prevalence and disease spectrum of extracoronary arterial abnormalities in spontaneous coronary artery dissection. *JAMA Cardiol*. Published online November 24, 2021. doi:10.1001/jamacardio.2021.4690

eTable 1. Comparison of General and Coronary Characteristics Between SCAD Patients With and Without FM

eTable 2. Prevalence and Severity of Arterial Tortuosity in Renal and Iliac Vessels of Patients With Spontaneous Coronary Artery Dissection Versus Controls

eTable 3. Prevalence and Characteristics of Fibromuscular Dysplasia and Other Extracoronary Abnormalities Detected by MRA and CTA in Patients Evaluated by Both Imaging Modalities

eFigure. Flow Chart of Patients From the UK SCAD Registry Included in the Current Analysis

This supplementary material has been provided by the authors to give readers additional information about their work.

eTable 1. Comparison of General and Coronary Characteristics Between SCAD Patients With and Without FMD

	**Patients without FMD N = 118**	**Patients with FMD N =55**	***P-value***
Women (%)	113/118 (95.8)	54/55 (98.2)	0.42
Age at diagnosis (years)	44.6 ± 7.7	44.5 ± 8.3	0.96
Body mass index (kg/m^2)	25.6 ± 5.2	28.5 ± 17.0	0.10
Smoking habit<br>Never/former smokers<br>Current smokers	 89/118 (75.4)<br>27/118 (22.9)	 35/55 (63.6)<br>19/55 (34.5)	 0.27
Treated hypertension	20/118 (16.9)	11/55 (20.0)	0.63
Type 2 diabetes	0/118 (0)	0/55 (0)	-
Treated dyslipidaemia	11/118 (9.3)	3/55 (5.5)	0.39
Type of Acute Coronary Syndromes at presentation of SCAD:<br>NSTEMI<br>STEMI<br>Cardiac arrest	 76/118 (64.4)<br>34/118 (28.8)<br>8/118 (6.8)	 29/55 (52.7)<br>20/55 (36.4)<br>6/55 (10.9)	 0.31
Percutaneous Coronary Intervention<br>Bailout Coronary Artery Bypass Graft	39/118 (33.0)<br>2/118 (1.7)	20/51 (39.2)<br>3/55 (5.5)	0.44<br>0.16
Angiographic features:<br>1. Coronary artery involved (%):<br>LM<br>LAD<br>LCX<br>RCA<br>2. Portion of the artery involved (%):<br>Proximal involvement<br>Mid involvement<br>Distal involvement<br>Branch involvement<br>3. Multivessel (%)<br>4. Multisegment (%)<br>5. Saw Class (%)<br>1<br>2a<br>2b<br>3<br>4<br>Recurrence (%)	 <br>8/118 (6.8)<br>89/118 (75.4)<br>34/118 (28.8)<br>15/118 (12.7)<br><br>27/118 (24.2)<br>53/118 (44.9)<br>64/118 (54.2)<br>36/118 (30.5)<br>18/118 (15.3)<br>44/118 (37.3)<br><br>17/118 (14.4)<br>53/118 (44.9)<br>22/118 (18.6)<br>9/118 (7.6)<br>17/118 (14.4)<br>9/118 (7.6)	 <br>4/55 (7.3)<br>33/55 (60.0)<br>21/55 (38.2)<br>14/55 (25.5)<br><br>9/55 (14.3)<br>25/55 (45.5)<br>24/55 (43.6)<br>18/55 (32.7)<br>9/55 (16.4)<br>20/55 (36.4)<br><br>6/55 (10.9)<br>26/55 (47.3)<br>10/55 (18.2)<br>6/55 (10.9)<br>6/55 (10.9)<br>7/55 (12.7)	 <br>0.91<br>0.04<br>0.22<br>0.04<br><br>0.33<br>0.95<br>0.32<br>0.77<br>0.85<br>0.91<br><br>0.96<br><br><br><br><br>0.45
Beighton score<br><4<br>≥4	 82/118 (69.5)<br>36/118 (30.5)	 38/55 (69.1)<br>17/55 (30.9)	 0.96
Migraines	59/118 (50.0)	24/55 (43.6)	0.56
PHACTR1<br>AA<br>AG	 53/114 (46.5)<br>55/114 48.2)	 20/49 (40.8)<br>23/49 (46.9)	 0.50<br>0.88

GG	6/114 (5.3)	6/49 (12.2)	0.12
Aneurysms	8/118 (6.8)	5/55 (9.1)	0.59
Arterial dissections	2/118 (1.7)	1/55 (1.8)	0.95
Tortuosity*	79/118 (66.9)	42/55 (76.4)	0.21

*defined as one or more curves ≥45°

SCAD: Spontaneous Coronary Artery Dissection; FMD: fibromuscular dysplasia.

eTable 2. Prevalence and Severity of Arterial Tortuosity in Renal and Iliac Vessels of Patients With Spontaneous Coronary Artery Dissection Versus Controls

**Tortuosity evaluation**	**Patients with SCAD N= 173**	**Controls N=41**	***P*-value**
	Renal arteries		
Right Renal artery			
Straight (%)		36/41 (87.8)	0.49
Tortuosity Index	158/173 (91.3)	21.0 (1/41)	0.69
Number of curves ≥45°	14.0 ± 5.7	1.0 (1/41)	0.78
Mean angle	6.9 ± 21.4	62.0 (1/41)	0.45
	54.9 ± 8.7		
Left Renal artery			
Straight (%)	161/173 (93.1)	40/41 (97.6)	0.28
Tortuosity Index	29.1 ± 9.3	25.0 (1/41)	0.71
Number of curves ≥45°	1 (1/173)	-	-
Mean angle	70.1 ± 13.6	-	-
	Iliac arteries		
Right Common Iliac artery			
Straight (%)	163/173 (94.2)	41/41 (100)	0.12-
Tortuosity Index	17.0 ± 7.4	-	-
Number of curves ≥45°	1 (1/173)	-	-
Mean angle	56.8 ± 12.6	-	
Right External Iliac artery			
Straight (%)	170/173 (98.3)	41/41 (100)	0.49
Tortuosity Index	16.3 ± 17.2	-	-
Number of curves ≥45°	1.5 ± 0.7	-	-
Mean angle	68.5 ± 29.0	-	-
Left Common Iliac artery			
Straight (%)	155/173 (89.6)	38/41 (92.7)	0.55
Tortuosity Index	16.1 ± 9.5	19.7 ± 8.6	0.55
Number of curves ≥45°	1.1 ± 0.3	1 ± 0	0.71
Mean angle	61.2 ± 14.7	68.5 ± 24.8	0.56
Left External Iliac artery			
One or more curves	171/173 (98.8)	41/41 (100)	0.40
≥45° (%)	26.8 ± 34.0	-	-
Tortuosity Index	2 (1/173)	-	-
Number of curves ≥45°	67.5 (1/173)	-	-
Mean angle			

eTable 3. Prevalence and Characteristics of Fibromuscular Dysplasia and Other Extracoronary Abnormalities Detected by MRA and CTA in Patients Evaluated by Both Imaging Modalities

FMD characteristics	MRA N=43	CTA N=43	*P-value*
Prevalence of multifocal FMD (%)			
At least one arterial bed	15/43 (34.9)	15/43 (34.9)	1.00
At least two arterial beds	7/43 (16.3)	7/43 (16.3)	1.00
Three or more arterial beds	1/43 (2.3)	1/43 (2.3)	1.00
Prevalence of FMD lesions in each vascular bed			
Cervical	6/43 (14.0)	8/43 (18.6)	0.56
Renal	10/43 (23.3)	7/43 (16.3)	0.42
Aorta	0/43 (0)	0/43 (0)	-
Visceral	2/43 (4.7)	3/43 (7.0)	0.65
Iliac	5/43 (11.6)	5/43 (11.6)	1.00
Intracranial	0/43 (0)	1/43 (2.3)	0.31
Aneurysm (%)	7/43 (16.3)	7/43 (16.3)	1.00
Intracranial aneurysm (%)	2/43 (4.7)	2/43 (4.7)	1.00
Arterial dissection	1/43 (2.3)	1/43 (2.3)	1.00

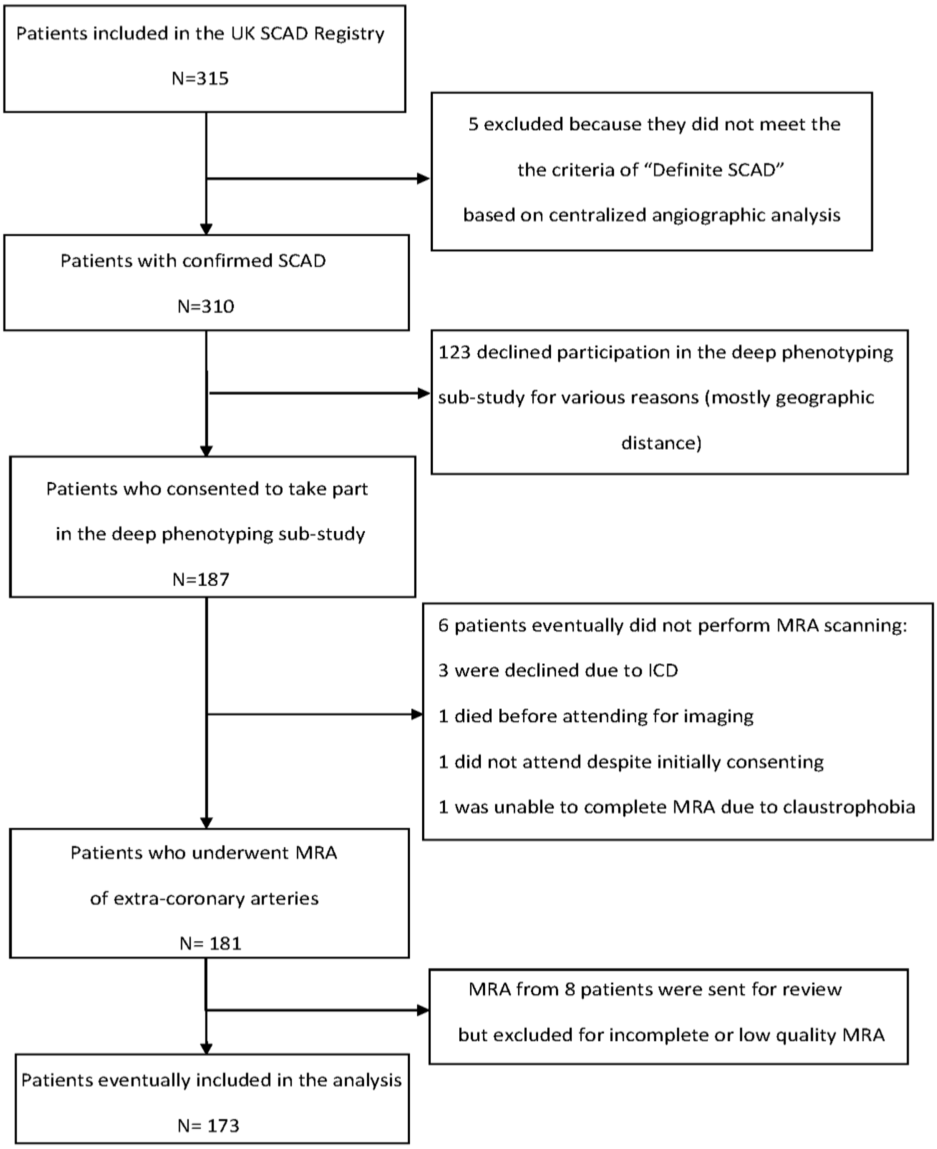

Figure S1: Flow-chart of patients from the UK SCAD Registry included in the current analysis.

eFigure. Flow Chart of Patients From the UK SCAD Registry Included in the Current Analysis
